# Advancing a food loss and waste bioproduct industry: A critical review of policy approaches for application in an Australian context

**DOI:** 10.1016/j.heliyon.2024.e32735

**Published:** 2024-06-09

**Authors:** Hannah Churton, Bernadette K. McCabe

**Affiliations:** a Centre for a Waste Free World, Faculty of Business and Law, Queensland University of Technology, Brisbane, QLD 4001, Australia; b Centre for Agricultural Engineering, University of Southern Queensland, West Street, Toowoomba, QLD 4350, Australia; c Fight Food Waste Cooperative Research Centre, Wine Innovation Central Building Level 1, Waite Campus, Urrbrae, SA, 5064, Australia

**Keywords:** Bioeconomy, Circular economy, Biorefining, Food waste, Bioproducts, Policy

## Abstract

Food loss and waste (FLW) contains an abundance of nutrient components that can be extracted and converted into valuable bioproducts through biorefining (e.g., pharmaceuticals, cosmetics, nutrients). Australia has identified bioproducts from a FLW feedstock as one avenue through which it can meet its commitment to UN Sustainable Development Goal Target 12.3, aiming to halve food waste by 2030. An industry for bioproducts in Australia is, however, nascent and will require targeted and sustained policy intervention to advance in line with the production targets it has set to meet Target 12.3. The aim of this critical review is threefold. Firstly, it draws on the research literature to identify barriers to advancing a bioproduct industry from FLW. Secondly, it constructs a taxonomy of policies available to overcome these barriers and support industry development. Finally, it applies the taxonomy to established policy settings in Australia (examining both national settings and Queensland state settings) and the European Union (EU), where the industry and associated policy is more mature. Australia has few national policies directly targeting a bioproduct industry. A comparative assessment of policy settings allows this review to identify lessons Australia can draw from the EU experience as it advances its own industry. Findings demonstrate a complex and fragmented policy landscape. Key recommendations from the literature emphasise the need to establish coordinated strategic instruments; target research and development opportunities for optimised, sustainable processes; and implement appropriate incentives to establish a ‘level playing field’, as technology readiness increases. The critical requirement for policy stability and coherence, flags the need to lay groundwork policy in this area as a priority.

## Graphical abstract

**Figure:**
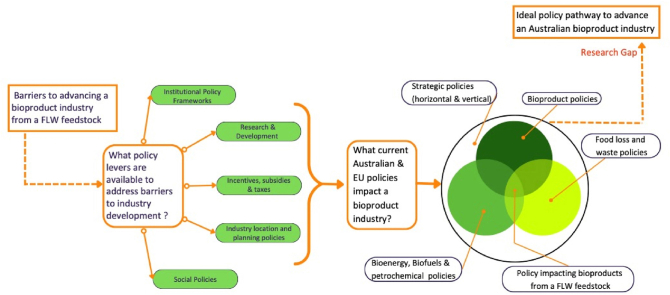

## 1 Introduction

Food loss and waste (FLW) contains highly variable nutrient and chemical characteristics – including valuable essential oils, pectin, vitamins, minerals, trace elements and bioactive compounds such as phenolic compounds, glucosinolates, flavonoids, and carotenoids [[1], [2], [3], [4], [5], [6], [7], [8]]. These characteristics have broad application in bioproducts, including pharmaceuticals, cosmetics, vitamins, chemicals, materials and ‘plastics’.

Converting FLW into bioproducts is an emerging industry cutting across multiple sectors key in the complex transition to a sustainable economy (agriculture, food systems, biotechnology, circular economy and bioeconomy). Policy will play a key role in advancing a bioproduct industry, driving desired economic, environmental and social outcomes, navigating externalities and maintaining a strong science-policy interface in the broader transitional context. Reduced FLW is a key desired outcome of a FLW bioproduct industry, addressing a major global issue that has clear and time-sensitive objectives.

One third of all the food the world produces is estimated to be lost or wasted [9]. The economic, environmental, and social costs of this waste are significant. Annually, it amounts to 3.3 gigatonnes of carbon dioxide (CO2) equivalent (approximately 8–10 % of greenhouse gas emissions) and it is attributable to the use of 250 km^3^ of blue water and 1.4 billion hectares of the world's agricultural land. The economic cost of this waste amounts to US$2.6 trillion annually [10,11].

The FLW problem is prioritised in United Nations Sustainable Development Goal (SDG) Target 12.3 aimed at halving per capita global food waste at retail and consumer levels and reducing food losses along production and supply chains by 2030 [12]. According to official guidance on interpreting SDG Target 12.3 [13], achieving the target requires 1) preventing food from leaving the human food supply chain or 2) diverting that which does leave the human food supply chain toward high-value destinations. A ‘high-value destination’ is comprised of one of only two potential pathways: 1) animal feed or 2) bio-based materials/biochemical processing, the latter occurring through biorefining processes, the subject of this paper.

Australia has committed to halving its food loss and waste by 2030 in line with SDG Target 12.3. The National Food Waste Strategy Feasibility Study [14] identifies ‘nutrient extraction’ (by means of product-driven biorefining) as one of 23 recommended interventions required to meet the target. According to the study, if the recommended intervention around nutrient extraction is implemented, extracting nutrients from food waste will contribute 163,839 tonnes towards Australia halving its food waste in 2030, compared to only 45,511 tonnes if it maintained the status quo [14]. Meeting the Feasibility Study target will therefore require a concerted increase in production. This gives rise to the question of how Australia will manage the increase.

Australia's National Food Waste Strategy (2017) [15] and the associated Feasibility Study acknowledge that policy will be a key driver in meeting food waste reduction targets, but there is limited research on the policy approach that would best serve Australia with respect to increased product-driven biorefining. Available literature focuses predominantly on policies governing the transition to a bioeconomy generally [e.g., 15,16]. Alternatively, given bioeconomic strategies typically had an early focus on bioenergy and biofuels, research literature examines policies effecting these specific subsets of the bioeconomy. Previous reviews in either the fields of FLW or biorefining have not examined policy impacts on the bioproduct industry as a discreet industry, as it applies generally or in an Australian context. This paper's main contribution is to map the full spectrum of policies available to Australia to exploit product-driven biorefining for the purposes of meeting SDG Target 12.3.

This review is structured in three parts. Section 2 is comprised of a theoretical evaluation of the FLW biorefinery concept and provides a synopsis of the current industry status of bioproducts from FLW, outlining key barriers to independent growth of an industry. Relevant studies targeting FLW bioproducts are scarce. The literature typically discusses barriers in the context of cross-cutting sectors – e.g., food waste, biorefining, bioeconomy or circular bioeconomy. This review draws on the breadth of sectors studies in the literature to identify barriers specific to product-driven FLW biorefining. Section 3 establishes a taxonomy of potential policy levers available to address barriers, creating a tool to analyse FLW bioproduct policy pathways. Like Section 2, the taxonomy is compiled from studies analysing cross-cutting sectors from which policy levers relevant to product-driven FLW biorefining are identified.

Sections 4 compares policy frameworks in Australia and the EU against the established taxonomy. To better capture the Australian context, this review draws on policies at national level and Queensland state-level. A comparative assessment carries two functions: 1) to critically examine how policies in practice compare to recommended pathways for developing a bioproduct industry broadly; and 2) to explore how Australia might learn from comparative frameworks to advance its own industry. Relevant policies are fragmented across sectors and Section 4 examines grey literature to identify policies in different sectors that directly target or impact the bioproduct industry in each jurisdiction. Policy is created at different levels of government and the multitude of states and regions within each jurisdiction determines that this grey literature review is not comprehensive. The analysis and conclusions of this review (Section 5), focuses on the role of multilateral and national governments in effecting policy change.

## 2 Bioproducts from food loss and waste: an industry context

### 2.1 Bioproducts from food loss and waste defined

**Fig. 1:**
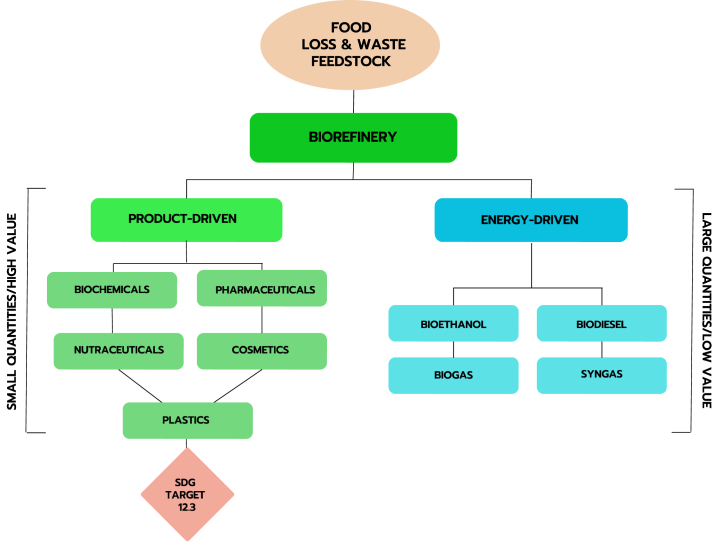
Product-driven and energy-driven biorefining processes.

Bioproducts are derived through biorefining – the sustainable and synergetic processing of biomass into marketable food and feed ingredients, chemicals, materials and energy [18].^1^ Biorefining processes are categorised in the literature as either energy-driven or product-driven. Energy-driven biorefining generally produces large quantities of low-value energy products (e.g., power, heat and fuels) as opposed to product-driven biorefining which generally produces small quantities of high-value, non-energy bioproducts (e.g., chemicals, materials, plastics and pharmaceuticals) (see Fig. 1.) [18]. FLW can be used as a feedstock for both product-driven and energy-driven biorefining processes. In accordance with guidance on SDG Target 12.3, however, only product-driven biorefining is considered a ‘high-value destination’ for the purposes of reducing food waste [13]. Given this review considers bioproducts through the lens of FLW reduction, its focus is product-driven biorefining.

FLW product-driven biorefining typically extracts unique chemical properties from specific feedstocks (e.g., essential oils from oranges, phenolic compounds from olive pomace). It therefore relies on a homogenous feedstock and is primarily derived from FLW at the production and manufacturing sectors of the food supply chain. It is notable that ‘food loss’ and ‘food waste’ are distinguished in the literature according to where waste occurs along the supply chain. Food loss occurs from harvest up to, but not including, the retail sector; with food waste occurring at retail and consumption [11]. Therefore, while this review adopts the term ‘FLW’, it is predominantly concerned with food *loss*.^2^

### 2.2 Current industry status

The technical feasibility of FLW as a feedstock for product-driven biorefining is well researched and showcases a wide range of FLW containing highly variable chemical characteristics with broad application in bioproducts [2,5,[20], [21], [22], [23], [24]]. Although the precise measure of available feedstock suitable for product-driven biorefining has not been examined in the literature (see Section 2.2.2), the Food Waste Index Report (2021) measured global food losses at 13.2 % [25]. In Australia horticultural losses are estimated at 18–22 % [26].

Transitioning away from petrochemical refining to biorefining is key component in the broader transition to more sustainable, circular bioeconomies. The global bioeconomy is projected to grow rapidly in the near future. In the European Union, for example, the Bioeconomy Footprint increased by +23 % between 2010 and 2020 [27] and bio-based chemicals are expected to reach a share of 15 % of the global chemical market by 2025 [28]. Product-driven biorefining is increasingly presented in emissions reduction, bio- and circular economic governance frameworks, a trend aligning with global objectives towards sustainable practices, most notably the Sustainable Development Goals [12] and the Paris Agreement [29].

Despite market growth and potential across the bioeconomy broadly, product-driven biorefining is an emerging industry globally and in Australia the industry is nascent. As of 2018, there were only 14 projects for bioproducts in total (with a further 11 commissioned), including projects for biofuels [30]. There are limited examples documenting bioproducts from a FLW feedstock in the literature – the most recent baseline data indicates only the wine making and livestock industries divert waste to ‘bio-based materials/biochemical processing’ [31]. This can be compared to more advanced (albeit still considered emerging) industries, such as the EU where there are 788 biorefineries integrating the production of products and energy and over 200 biorefineries utilising a waste feedstock [32]. Globally, there are relatively few product-driven biorefineries from a FLW feedstock at commercial scale. A clear business case for production and manufacturing stakeholders (e.g. farmers, processors) to reduce their FLW through product-driven biorefining is therefore largely lacking [11]. Section 2.3 reviews literature that identifies the unique barriers and challenges to advancing an FLW biorefining industry for commercial operation.

### 2.3 Defining barriers & challenges

Barriers and challenges were identified by employing a scoping review conducted via SCOPUS journal search. The initial search revealed few studies specifically highlighting the challenges of advancing an industry for FLW product-driven biorefining. Searches employing broader criteria found that barriers are more frequently explored in the context of product-driven biorefining more broadly or in the context of the barriers to ‘bioeconomy’, ‘circular economy’ or ‘circular bioeconomy’ generally. Following the application of these broader search terms, data was organised across themes that could be applied to FLW biorefining specifically, identifying eight key barriers: 1) technology; 2) feedstock availability and logistics; 3) economic viability; 4) market demand; 5) environmental impacts; 6) social impacts; 7) level playing field; and 8) policy stability and coherence. Each of these is explored in more detail below.

#### 2.3.1 Technology

Advanced, integrated biorefineries are widely considered key to the economic feasibility of FLW treatments [5,17,23,[33], [34], [35]]. Integrated FLW biorefining combines different technologies, allowing for multiple feedstock streams and the recovery and production of multiple high-value and low-value products in economies of scope [23,36,37]. This accommodates 1) the full utilization of feedstocks, maximising the use of waste resources through cascading uses of food waste streams; 2) more resilience for food waste biorefineries in coping with potential feedstock availability issues; 3) the diversification of revenues by covering multiple markets/niches; 4) synergy effects of different technologies; 5) the sharing of human resources and equipment; and 6) potential achievements of energy self-efficiency e.g., biogas production. Key technologies, however, remain in the R&D, pilot and demo-phases [5,18,[38], [39], [40]].

#### 2.3.2 Feedstock availability & logistic

Many types of food wastes are generated seasonally, are difficult to store and can deteriorate quickly, which presents challenges to the generation of a sufficient quantity and quality of the required feedstock for industrial application [5,38,41]. The location of a biorefinery is therefore significant to the feasibility of an operation – storage and deterioration challenges not only place limits on the feasibility of transporting feedstock, but can have an important influence on operational viability, accruing economic and environmental costs [23,38,[42], [43], [44]]. Locating biorefining facilities so they are proximate to the source of feedstock can go some way to mitigating those challenges. Production and manufacturing sectors of the food supply chain are, however, typically located in regional areas, which present their own challenges, e.g. distance to potential markets and complex social impacts (see Section 2.2.5).

#### 2.3.3 Economic viability

The economic viability of any biorefinery is dependent on a broad range of variables, including: investment costs, feedstock cost and availability, biomass-to-product yield, energy efficiency, pre-treatment and production costs, location, supply chain configurations, existence of end-product market, market prices, economies of scale and biomass market impacts [23,35,[43], [44], [45]]. The novelty of advanced integrated biorefinery technologies and associated feedstock and logistics issues means the economic viability of FLW bioproduct biorefineries at scale is largely untested and uncertain [5,23,43,44,[46], [47], [48], [49]]. Highly-capital intensive biorefineries therefore continue to represent major risks for investors [35,50,51].

#### 2.3.4 Market demand

Major hurdles to capturing market share from conventional product manufacturers remain for bioproducts [23,37]. Petroleum-based products retain advantages associated with conventional product incumbency (e.g., brand recognition, established distribution channels, capturing market share based on price point). Compared to conventional products, many bioproducts are still new to market and consumer knowledge remains quite low [52,53]. Introducing bio-based alternatives to existing products can benefit from an already established market, avoiding the costs of extensive marketing to create demands for new products. Reducing costs and driving customer behavioural change towards sustainable choices will nonetheless play a crucial role in building market demand for bioproducts [23].

#### 2.3.5 Environmental impacts

Compounding the challenge of economic viability, is the bioeconomic promise of sustainable economic growth, that assumes profitability won't come at an unacceptable environmental or social cost. Sustainability is, however, not self-evident and how a ‘balance’ between potentially competing objectives should be struck is unresolved in the literature. Overall, political discourse on bioeconomy to date has had a strong focus on the economic dimension, with environmental and social considerations remaining peripheral [54,55].

Environmental impacts of the process are dependent on the individual methods and processes adopted [56]. Criteria for establishing environmental efficiency are varied and extensive with no consistent method for assessing the environmental sustainability of product-driven biorefineries. This has precluded conclusions on which valorisation pathways are more sustainable [2,23,38,52]. Available studies show promising environmental outcomes overall but demonstrate that the environmental impacts of valorisation can outweigh the benefit derived from reusing a stream of food waste [36,[57], [58], [59], [60]].

#### 2.3.6 Social impacts

The bioeconomy seeks to influence almost all areas of social life, the impacts of which are often deeply rooted in the cultural habits of society and are, therefore, extremely difficult to change [16]. Social barriers specific to advancing a bioproduct industry include: competition for feedstock, lack of skilled workforce, limited public perception and awareness, and changes to distribution of income and economic possibility [5,37,[61], [62], [63], [64]].

#### 2.3.7 Level playing field

A lack of level playing field is a critical barrier to market and industry growth for bioproducts [17,[65], [66], [67]]. Firstly, petrochemical refining continues to enjoy wide policy and financial support globally [17,68,69], contributing to a bioproduct industry's inability to be cost-competitive with fossil-fuel counterparts. Secondly, there is typically a lack of level playing field within the biorefining industry itself, with policy support for bioenergy and biofuels much greater than for bioproducts. Bioenergy and biofuels not only receive high support in R&D, pilot and demonstration plants, but also receive strong ongoing support during commercial production (e.g., quotas, tax incentives, and green electricity regulations) [17,65]. By comparison, product-driven biorefining has been relatively overlooked by policy makers. If the bioenergy and biofuels markets are more attractive on account of related incentives and support, biorefinery development will disproportionately focus on these sectors, at the expense of bioproduct industry development.

#### 2.3.8 Policy stability & coherence

There is a lack of policy coherence, stability and certainty present for the bioproduct industry, all of which are key to investment [63,[70], [71], [72]]. Governments play a critical role in ensuring policy stability and coherence, but as an emerging industry, they have not had the benefit of time to develop the requisite policy stability in the sector - the OECD recommending a timeframe of 15–25 years of coherent policy to develop requisite stability for market certainty [72]. Moreover, policymakers are challenged to keep pace with rapidly evolving industry developments. Aligning domestic policy with government commitments to international instruments (e.g. SDGs and emissions reduction targets) would assist mitigate some of these challenges as would adopting a range of adaptable, complementary policy combinations (over, for example, less-flexible, command and control instruments) [73]. Available literature points to the need for responsive, flexible, integrative policy-making across sectors, avoiding sectoral ‘silos’, to balance competing economic, environmental and social demands or achieve synergies [70,71,74]. Balancing tensions, uncertainty, and vagueness around policy goals, ensures this is highly complex.

## 3 A public policy approach to advancing the bioproduct industry

Academic analysis of the bioproduct industry is in broad agreeance that the industry will not overcome the barriers outlined in Section 2 without policy intervention [16,60,68,75,76]. Policy levers potentially impacting a FLW bioproduct industry involve a complex web of direct and indirect policy domains, spanning global challenges that include biomass, sustainability and climate change as well as highly localised issues that include regional development, waste management, and labour force preparedness [17]. This review adopts a “smart regulation” approach to policy making, as advocated by Gunningham and Sinclair [77], a theory which posits that implementing multiple complementary combinations of instruments will better deliver on purported policy goals than relying on single policy instruments. This study, therefore, investigates a broad spectrum of ‘policy’, encompassing all forms of government intervention that promote or inhibits the bioproduct industry - including strategies, legislation, regulatory measures, and funding priorities. An exhaustive review of individual policy levers that potentially impact the FLW bioproduct industry is beyond the scope of this review. Rather, section 3.1 draws on research literature to identify the overarching and defining policy levers that go to directly targeting the challenges highlighted in Section 2.

### 3.1 A taxonomy of policy levers for advancing a FLW bioproduct industry

Limited consideration of policy frameworks targeting FLW bioproduct biorefining is available in the literature. This section therefore reviews literature that considers policy levers relevant to the bioproduct industry, the biorefining industry more broadly, the bioeconomy or the circular economy, and seeks to identify policy levers relevant to FLW bioproduct biorefinery specifically. The review was conducted via a SCOPUS journal search, applying the following search strings:

“food waste” AND “biorefining” OR “biorefinery” AND “high-value bioproducts” OR “high-value bio-based products” OR “biochemicals” OR “bio-based chemicals” OR “biomaterials” OR “bio-based materials” OR “value-added products” OR “bioeconomy” OR “circular bioeconomy” or “circular economy” AND “policy” OR “governance”, with the search statement expanded to include “food waste” in ‘all fields’.

Following the database search, we reviewed the results for inclusion or exclusion. Our criteria for inclusion of literature within our review were: (1) journals that were peer-reviewed, (2) material published in English, (3) material published since 2018 to limit our analysis to relatively recent developments in the topic; and (4) material that analysed policy.

Records were filtered by title and abstract to identify literature relevant to FLW product-driven biorefining. As relevant policies emerged, studies were grouped into themes and further specific search statements were applied to capture the full scope of relevant literature. We also reviewed the reference lists of articles and included relevant articles from those lists.

**Fig. 2:**
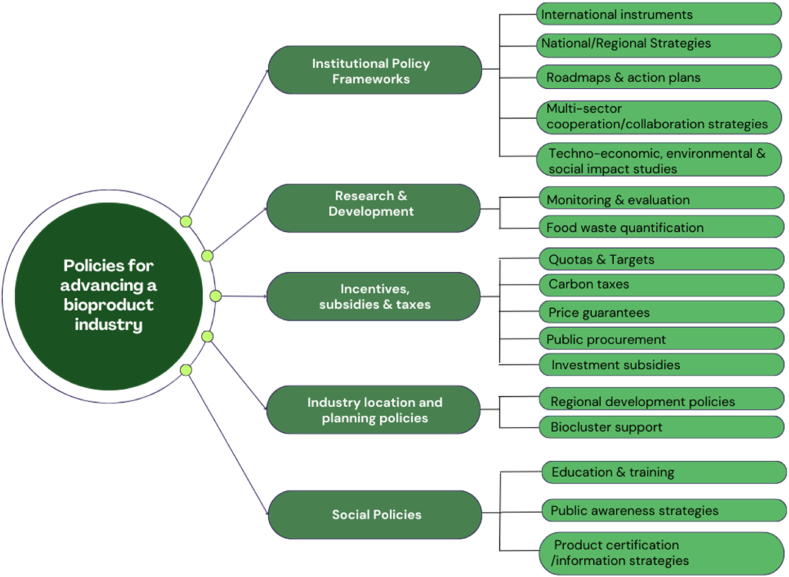
Taxonomy of available policy levers to advance a FLW bioproduct industry.

The review revealed five key policy categories, that can each be broken down into multiple policy levers as outlined in the taxonomy at Fig. 2. Individual policy levers are explained in detail below, including how they stimulate or inhibit the bioproduct industry generally and the impacts this might have on an emerging FLW bioproduct industry specifically.

#### 3.1.1 Strategic policy frameworks

FLW bioproduct biorefining is fragmented across multiple policy domains including (but not limited to) FLW, biorefining, climate and agro-food policy. Strategic frameworks attempt to provide overarching vision and intent to policy and play an important role in bringing coherence across policy domains and stability to industry [71,72,78]. Research highlights, however, that frameworks alone are insufficient for that purpose and must be supported by clear implementation and coordination mechanisms. Examining biorefining broadly, the [72] suggests a two-pronged strategy: 1) a biorefinery roadmap; and 2) an enablement-oriented action plan, accompanied by a national/regional leadership council of public and private actors to make sure that milestones are being met. Beyond meeting milestones, the OECD [72] considers careful national/regional and inter-agency leadership, coordination and collaboration at regional, state and federal levels supporting strategy critical to establishing stability and coherence across policy domains.

#### 3.1.2 Research & development grants

The need for ongoing research and development (R&D) to increase technology readiness of integrated biorefineries and prove sustainability at a commercial scale is frequently cited in the literature [5,23,34,35,[38], [39], [40],^43^,[79], [80], [81], [82], [83], [84]]. Studies consider that identifying optimal FLW bioproducts and processes with consideration for technical, economic, environmental and social components key to this process.

Different methodologies have been adopted across the research literature to assess processes, including techno-economic analysis, life cycle costing, life cycle assessment and social life cycle assessment [82,85]. Methodologies are not standardised, an issue which warrants closer R&D consideration in and of itself. Developing standardised modelling frameworks and criteria would allow for the systemisation of cases and the comparison of results [82,86]. Multiple studies assert that further R&D should be devoted to the development of integrated analyses for biorefining processes, allowing for the systematic analysis of the relationships between technical, economic, social and environmental performance and provide more data for trade-off analysis [79,81,82,[86], [87], [88]]. Research analysing the broader bioeconomy also highlights the need for the development of comprehensive frameworks for measuring and evaluating industry progress [16,[89], [90], [91], [92]]. Monitoring frameworks are a crucial component of R&D, highlighting synergies and trade-offs and supporting the identification of areas in need of policy intervention [92].

Critical to assessment processes for FLW bioproduct biorefinery specifically, is the quantification and reliability of FLW available as a feedstock [39,43,46]. Cristobal's 2018 study determined that 1) the available amount of FLW feedstock and 2) the location the waste was generated, influenced the economic sustainability of the biorefinery and were crucial factors in assessment [43].

#### 3.1.3 Incentives, subsidies & taxes

Incentives, subsidies and taxes – economic policy instruments – are an important means by which government can encourage investment in high-risk new industries. Economic policy instruments are also advanced in the literature as key mechanisms for creating a level playing field [66,93]. A level playing field for bioproducts from a FLW feedstock would not only allow bioproducts to compete with its bioenergy and biofuels counterparts but could support circular principles by encouraging the allocation of FLW feedstock to the ‘highest value use’ within a cascading framework [94,95].

Typically, there is a disproportionate level of economic policy support for bioenergy and biofuels, where carbon pricing and mandatory quota systems are common place, compared to bioproducts where equivalent measures haven't been extended [52,66,67,74]. This can, however, be attributed in large part to a less developed bioproduct industry. In their examination of policy instruments suitable for an advanced biorefining industry, Hellsmark and Söderholm [96] highlight five phases of innovation (in ascending order of development: concept development; demonstration; niche market; commercial growth; maturity). They position advanced/integrated biorefineries in a late demonstration phase, working towards a niche market phase. Carbon pricing and mandatory quota systems were identified as relevant policy instruments for technologies starting in the growth phase, but neither were considered sufficient instruments for stimulating investment in earlier phases: carbon pricing on its own did not provide enough incentive for investments in the first commercial-scale plants; quotas were only considered effective for diffusing relatively mature technologies, favouring established actors with existing production, distribution systems, and end-use markets and cases where there is a good understanding of the opportunities for up-scaling.

In the niche market phase, Hellsmark and Söderholm determine that creating stable conditions and long-term stability is of paramount importance, requiring policy that can create a market, support technology development and allow for the first commercial-scale plants to be built [96]. Appropriate policy incentives for a bioproduct industry at its current stage of development were identified as public procurement, price guarantees and investment subsidies (coupled with carbon pricing to stimulate wider diffusion). These levers provide a temporary protected space in which the technology can develop with less direct competition. A study of existing regulatory and standardisation barriers to bioproduct uptake by Ladu and Vrins [67] adds that public procurement should be complemented by sustainability certification for all products, and corresponding standards and labels including clear information on how products should be disposed of and subsidies for administrative costs for producers.

#### 3.1.4 Industry location and planning policies

A strong narrative has developed within bioeconomy discourse around the potential for ‘bioclusters’ to solve feedstock logistics and transportation challenges [97,98]. Bioclusters entail manufacturing in small- and medium-scale integrated biorefineries in close proximity to the feedstock, thereby minimising transport and logistics challenges and maximising supply chain availability and quality. Bioclusters also offer the additional advantage of localising the whole production value chain within the same region, promoting innovativeness, productivity, regional economic development, employment, and business competitiveness. The biocluster concept has become central to many national bioeconomy strategies and there are high expectations of the contribution of bioclusters to the transition away from the use of fossil fuels while fostering innovation and rural development [16,63,97]. Philp [17], however, highlights, that the biocluster vision can be at odds with the current reality of massive petrochemical economies of scale and Ayrapetyan & Hermans [97] note negative social (e.g., social cohesion, justice, health, labour rights) and environmental effects (e.g., ecosystems, habitat, biodiversity) that bioclusters risk both inside and outside their region. Decisions on function and location of a biocluster are not simple – economic, environmental and social effects on the immediate and broader community must be well-understood and accounted for by governments and decision-makers to mitigate negative impacts [72], reinforcing the need for robust and holistic R&D to assess these effects.

#### 3.1.5 Social change policies

Significant and multiple levels of social policy support is required to advance a bioproduct industry. This includes: 1) public awareness campaigns targeting the benefits of buying bioproducts; 2) a certification system to help consumers identify bioproducts; 3) monitoring and evaluation of the impacts of bioclusters on regions; and 4) labour education, training and workforce preparedness [37,62,63,90]. Regarding the latter, Delebecque and Philp [62] conclude that training for bioeconomy readiness calls for a paradigm shift in how education is structured, including multi- and interdisciplinarity across systems thinking, strategic planning, and evaluating environmental, social, and economic performance. The literature recommends a broad spectrum of disciplines be targeted in this process, including farmers, natural resource managers, scientists, social scientists, engineers, managers, construction workers, economists, analysts, educators, regional actor networks, market and business specialists [37,63].

## 4 A comparative overview of Australian & EU policy settings

This section overlays the taxonomy developed in Section 3 with current biorefining policy settings in Australia and the EU – the EU having been selected for its industry's relative maturity and the body of work available in the literature for comparative purposes. This review relies primarily on grey literature to examine policy settings against the theoretical policy pathways provided in Section 3's taxonomy and provide a basis from which to assess how Australia might draw on the comparative experience in the EU to expedite its own industry advance and meet its commitment to SDG Target 12.3. On account of the significant indirect impacts that policy settings targeting bioenergy and biofuels sectors have on a FLW bioproduct industry, this section considers policies across all biorefining sectors. The strong interrelationship between biorefining and bioeconomic and circular economic policies also warrants consideration.

In Australia, state governments are largely responsible for setting policy across the relevant policy domains (FLW, biorefining, climate and agro-food policy). The national government takes responsibility for strategic policy (on issues that have a national impact), national coordination, managing international agreements and funding support. Limited policy intervention targeting bioproduct industry development has occurred at national level in Australia and distinct and differing policy settings have been adopted across state jurisdictions. Following the 2022 federal election, the Australian Government has undergone a substantial step-change in relation to broader and related policy issues, most notably energy, emissions reduction, and climate action. The enduring effect of these changes is yet to fully materialise but, at a minimum, the shift is expected to have significant indirect implications for the biorefining industry broadly.

By comparison, ‘bioeconomy’ has been formally embedded in EU policy settings since 2012, with a complex system of policies across multiple policy domains evolving in the intervening decade that directly target product-driven biorefining, including from a FLW feedstock. Notwithstanding a comparatively mature industry, the bioproduct industry is still relatively novel even in the EU, with associated policy frameworks considered nascent or emerging. This section compares EU policy settings (acknowledging the EU's inherently multilateral nature) with national policy settings in Australia. While it doesn't provide the scope to examine all state government jurisdictions, it explores policy settings established in Queensland to exemplify the vertical interaction of policy across national and state jurisdictions.

### 4.1 Strategic policy frameworks

Strategic policy instruments targeting product-driven biorefining from FLW at national level in Australia are confined to the waste domain. The *Recycling and Waste Reduction Act (2020)* [99] emphasises circular economy as a central objective of waste reduction, but does not make explicit provision for FLW. The National Food Waste Strategy [15] and National Food Waste Strategy Feasibility Study [14] are the primary frameworks targeting Australia's commitment to SDG Target 12.3, defining FLW as ‘food that does not reach the consumer or reaches the consumer but is thrown away.’ Notably, the Feasibility Study specifically identifies ‘nutrient extraction’ (by means of product-driven biorefining) as one of 23 recommended interventions required to achieve the target. Paradoxically, it also explicitly excludes the diversion of FLW to biomaterials as a pathway for contributing to Target 12.3, despite the process reintegrating FLW into the food supply chain – this contradicts official guidance on interpreting Target 12.3. In 2023, Australia's National Science Agency, CSIRO, published a roadmap towards a more sustainable future for Australia's food, which includes a 2050 goal of zero unavoidable food waste, including through processes of transforming FLW into value-added products [100].

Australia hasn't developed broader national strategic instruments for a bioeconomy. Applying circular economic principles is the key aim of the *National Waste Policy: Less Waste More Resources* (2018) [101] and the accompanying Action Plan (2019) [102] (as jointly agreed by federal and state governments). While no specific reference to bioproducts (from a FLW feedstock or otherwise) is made within these instruments, recycling in accordance with circular economy principles is emphasised. Strategic frameworks within the biorefining industry apply to bioenergy and biofuels only. In support of more ambitious climate action and emissions reduction targets, Australia has recently introduced a host of policy strategies to fast-track energy transition, including, Rewiring the Nation Plan [103], the National Energy Transformation Partnership [104] and National Energy Performance Strategy [105]. The Bioenergy Roadmap (2021) [106] and Future Fuels and Vehicles Strategy (2021) [107] provide growth strategies for bioenergy and biofuels industries focused on enabling market opportunities, developing resources and building ecosystems. There are no equivalent strategies guiding policy decisions on bioproducts at national level.

In Queensland, a general waste hierarchy is embedded in the *Waste Reduction and Recycling Act 2011* [108], setting a clear legal foundation to guide policy. The Act legislates the promotion and facilitation of Queensland's transition to a circular economy, explicitly preferencing recycling of waste resources over recovery. This preference is mirrored across strategic frameworks targeting FLW and/or bioproducts more specifically. The *Waste Management and Resource Recovery Strategy* [109], the overarching framework for waste reform and implementation, governs multiple policies including the *Queensland Organics Strategy 2022–2032* [110] and accompanying action plan. This strategy highlights support for 1) the expansion of valorisation infrastructure to transform inedible by-products into valuable new products and 2) the development of biomanufacturing and biochemical processing capacity (including managing risks with market expansion). The *Energy from Waste Policy* [111], and the *Biofutures 10-Year Roadmap and Action Plan* [112], both make specific reference to the development of biotechnologies and the growth of a bioproduct industry from a waste feedstock. These policies expressly prioritise bioproducts and biofuels over bioenergy in line with circular principles. Bioproducts from a primary-production (waste) perspective are incorporated into the *Queensland Agriculture Strategy* (2013) [113], the *Low Emissions Agriculture Roadmap 2022–2032* [114], and the *Queensland AgTech Roadmap (2023–2028)* [115] each of which highlight new pathways for growth from alternative products, including pharmaceuticals and nutraceuticals. Details of how state strategies interact with federal strategy is unclear in the literature. Federal/state government coordination on adjacent issues is, however, emerging: in 2022, the Environment Ministers Meeting intergovernmental forum committed to achieving circular economy by 2030 [116], and a Circular Economy Ministerial Advisory Group established to advise government on making the transition [117]; the Energy and Climate Change Ministerial Council was also established in 2022, coordinating on priority issues in the energy and climate change sectors [118]. The Resource Recovery Reference Group, comprised of governments, non-government organisations, industry and business representatives, guides the implementation of the National Waste Policy Action Plan (2019) [119], exploring regulatory, financial and legislative options for achieving targets where necessary.

By comparison, the EU integrates its commitment to SDG Target 12.3 in strategic frameworks across multiple policy domains (economic, agriculture, primary production, waste). As a key process of the bioeconomy, biorefining processes are governed by all overarching frameworks guiding the transition to sustainable economies. Relevant overarching frameworks include: the European Green Deal (2019) [120] (providing a framework for economic sustainability crosscutting all sectors of the economy), the Bioeconomy Strategy (2018) [121] and the Circular Economy Action Plan (CEAP) (2015/2020) [122], each of which expresses the common aim of adding value to waste. More specifically, converting waste into bioproducts as a pathway for reducing food waste is one of six aims of the Farm to Fork strategy (2020) [123], a pathway mirrored in the amended Waste Directive (2008) [124]. The Common Agriculture Policy (2023–2027) [125] aims to reduce waste and more specifically, ‘improve the use of by-products, including their reuse and valorisation.’

The Bioeconomy Strategy focuses on three outputs: food and feed, industrial bio-based products and bioenergy. How these outputs are to be prioritised is not specified, although when read with EU circular economic guidelines, products ‘at their highest value’ are preferred (subject to balancing environmental and social externalities) [121]. It is noteworthy that, like Australia, the EU has specific strategic instruments dedicated to bioenergy and biofuels (e.g., Renewable Energy Roadmap, 2006; [126,127], with no strategic equivalent dedicated to bioproducts.

### 4.2 Research and development grants

In Australia, current research and development grants targeted at biorefining are exclusively focused on bioenergy and biofuels at national level. While not specifically targeting bioproducts, the End Food Waste Cooperative Research Centre TRANSFORM program provides funding to identify and prioritise valuable products from food waste streams [128]. CSIRO offers R&D grants of AUD10-50 k to start-ups and various funding opportunities for researchers [129] as does the Australian Research Council [130].

At state level, targeted grants for bioproducts have been available. In Queensland, grants opportunities have encompassed ‘biotechnologies’ broadly, thereby encompassing bioproducts. Queensland's Waste to Biofutures Fund (2019) [131], for example, targeted research collaboration for the commercialisation of Queensland's biofutures industry, in particular its capacity to produce biotechnologies encompassing bioproducts. The QLD-Germany Bioeconomy Collaborative Science Program [132] funds the development of technologies, including biotechnology, for the production of value-added products from a sustainably sourced biomass. The Biofutures Roadmap (2022) [112] emphasises the importance of government supported hubs, precincts and incubators to provide a strong focus on research outcomes and commercialisation. The planned Mackay Future Foods BioHub will explore horticultural waste as a feedstock for high-value bioproducts [133]. The Mackay Renewable Biocommodities Pilot Plant is a pilot-scale Research and Development integrated biorefinery for the conversion of cellulosic biomass into bioethanol and high-value biocommodities [133]. Monitoring frameworks to evaluate industry progress, synergies and trade-offs have not been established in Australia. Data on how funding levels for bioproducts compare to funding levels for biorefining or biofuels is likewise not available. Australia has mapped feedstock availability and location (including FLW), but for bioenergy uses only (much of which would not be suitable for high-value bioproduct purposes) [134].

The EU dedicates a far greater percentage of GDP to R&D than Australia and this likely reflects the level of funding committed to R&D in the bioeconomic sector (as a percentage of GDP, R&D expenditure stood at 2.23 % in 2022, compared to Australia's 0.49 % of GDP in 2022–23) [135,136]. Research and development (R&D) has been a key feature of the EU bioeconomy and associated strategies, which has evinced a strong focus on technological development. Horizon Europe [137] and the Circular Bio-based Europe Joint Undertaking [138] are key R&D funding instruments, with objectives including accelerating the innovation process and development of bio-based solutions; accelerating market deployment of innovative bio-based solutions; ensuring a high-level of environmental performance of bio-based industrial systems and strengthening and scaling up the EU bio-based sectors in all stages of the innovation cycle. Horizon Europe's Cluster 6 [139] specifically targets food, agriculture and bioeconomic innovation systems within that framework with a foreseen budget of EU9 billion [140].

While R&D is a focus of the bioeconomy broadly, programs typically treat biorefining as a single integrated category. Data relating to funding is not disaggregated between bioenergy/biofuel/bioenergy or around technologies that would support advancing the bioproduct industry (integrated biorefineries), making it difficult to determine how well the bioproduct industry is funded compared to other aspects of the bioeconomy. Project databases suggest a significant imbalance in R&D supporting bioproducts compared to those supporting bioenergy or biofuels. A basic word search in the EU's CORDIS database returned 1176 results for projects funded for ‘food waste bioproducts’ and 3830 results for ‘bioproducts’, compared to 7164 for ‘bioenergy’ and 9709 for ‘biofuel’ [141]. Search mechanisms don't provide an option to further filter results for high-value bioproducts from FLW, but it's nonetheless indicative of funding spread across different biorefining processes.

The EU Bioeconomy Monitoring System launched in 2020, catalogues and measures aspects of the bioeconomy, including bioproduct production, the uses of biomass, and the recovery of food waste (data on the diversion of food waste to bioproduction is not catalogued) [142]. It has also established multi-sector committees coordinating R&D for biorefining industries. Key to FLW biorefinery specifically, the recently released EU Biomass Flows [143] quantifies and maps food waste across all stages of the supply chain.

### 4.3 Incentives, subsidies and taxes

At national level, Australia offers economic support to the bioproduct industry through the National Reconstruction Fund [144], a funding initiative targeting projects that drive sustainable economic growth and the Industry Growth Program [145] provides funding to start-ups and early-stage business to commercialise. ‘Value-add’ in the agriculture and food sectors are listed as priority areas under both initiatives (alongside renewables). CSIRO's ‘Main Sequence’ funding program is aimed at addressing the ‘valley of death’ between research and commercialisation, including in the food sector [129]. The Research and Development Tax Incentive offsets costs to encourage industry to undertake R&D activities [146]. Under the Australian Carbon Credit Unit (ACCU) Scheme Australian [147], emissions reduced through the processing of organic waste that would otherwise end up in landfill are eligible for ACCUs. While notable, the current destination for feedstocks suitable for product-driven biorefining is typically animal feed or ploughing back in, not landfill.

Within the biorefining industry more broadly, the Renewable Energy Target incentivises the development of renewable energy power stations through a renewable energy certificate market. The current Australian Government has committed to reach a share of 82 % of renewable electricity by 2030 [148]. Various tax incentives exist to assist companies to fund energy efficiency projects (R&D tax incentive, instant asset write-off, accelerated depreciation deductions). Further, a wide range of rebates, funding, loan financing and other financing arrangements are available [149]. There is no federal government subsidy, tax credit or mandate supporting the production or use of biofuels. The industry does, however, benefit from excise tax credits [150].

In Queensland, there are a suite of incentives, subsidies and tax policies on offer directly targeting biorefining, including bioproducts. The Waste to Biofutures Fund provides co-funding for the purchase and installation of biorefinery plant and equipment; the Biofutures Industry Development Fund, funds well-advanced industrial biotech proponents to get large-scale projects through the final stage of financial due diligence to secure financing from investors; the Biofutures Commercialisation Program attracts national or international bioindustrial expertise to partner with Queensland researchers and/or businesses to scale-up and prototype new or improved industrial biotech and processes at the pilot or demonstration scale in Queensland; and the Biofutures Acceleration Program supports the development of new biorefinery projects in Queensland [151].

While not directly targeting bioproducts, incentives exclusively targeting biofuels or bioenergy are worth noting for their indirect (negative) impacts on the development of a bioproduct industry. Queensland has enforced a biofuels mandate since 2017, setting minimum requirements for the sale of biobased petrol and biobased diesel (*Liquid Fuel Supply Act 1984*) [152]. The Energy (Renewable Transformation and Jobs) Bill 2023 (QLD) sets ambitious renewable energy targets at 50 % by 2030 (80 % by 2035) [153]. Further, the *Waste Reduction and Recycling Act 2011* [108] and Waste Reduction and Recycling Regulation 2023 [154] impose a levy on waste to landfill. Although this levy is not expected to directly impact the bioproduct industry in Australia (on account of suitable feedstocks not typically destined for landfill), it does have potential to promote circular economic practices generally (a key objective of the Act) and may stimulate innovation in the use of biomass feedstocks more broadly for use in bioproducts.

Fossil fuels continue to enjoy significant subsidies. In 2021–22, it is estimated fossil-fuel subsidies cost Australia AUD 11.6 billion, the largest subsidy being the AUD 8 billion Fuel Tax Credit Scheme [155]. Subsidies for renewables do exceed that figure, the 2022-23 Australia budget committing AUD 25 billion to clean energy and renewables projects, although the degree to which this benefits bioproducts as a discreet industry is unclear. By comparison, the EU subsidised fossil-fuels by approximately EUR 50 billion in 2020 compared with EUR 81 for renewables [156].

Incentives and taxation instruments targeting bioproducts remain limited in the EU. It has not implemented public procurement policies or price guarantees for the bioproduct industry. Guarantees and direct loans have been available in the EU since 2020 through Horizon 2020 InnovFin (succeeded by the InvestEU Fund), the introduction of which was considered a major step in the portfolio of funding mechanisms [157]. The research literature has assessed this as a significant gap in the innovation cycle for Europe, impacting infrastructure, capability and capacity to commercialise results [72]. The primary financing mechanism prior to InnovFin was public-private partnerships involving matched funding. In addition, the EU deploys a targeted financial instrument - the Circular Bioeconomy Thematic Investment Platform - to de-risk private investments in sustainable solutions [121].

There are not currently any quotas or targets for the production of bioproducts in the EU, although the EU Biorefinery Outlook to 2030 [158] acknowledges this gap, recommending fossil-fuels reduction targets/mandates in EU chemical production be set, taxes on fossil inputs, and bans on selected fossil-based products as the industry developments. With respect to FLW, a Roadmap for Food waste Reduction Targets and legally binding targets to reduce food waste across the EU were expected to be imposed by the end of 2023, which, if introduced, may have flow-on effects for FLW bioproduct outputs. By comparison, ambitious targets have been established for the bioenergy and biofuels sectors. The Renewable Energy Directive II (RED II) [159] established a binding renewable energy target for the EU of 32 % by 2030. The REPowerEU Plan [160], however, sets out a series of measures to rapidly reduce the EU's dependence of Russian fossil fuels proposing an increased target of 45 %. RED II sets a 14 % renewable fuels target for the transport sector. Of further significance, although again targeting bioenergy and biofuels industries rather than bioproducts, are the improving regulatory standards for incentivising the use of waste over alternative biomass feedstocks (e.g., RED II, Indirect Land Use Change Regulation, Waste to Energy Directives and Sustainable Certification Schemes), which go some way to mitigating challenges arising from ‘food versus fuel’ considerations.

### 4.4 Industry location and planning policies

A general lack of national-level policies targeting bioproducts specifically, means there is no express reference to industry location and planning policies for a bioproduct industry in Australia. More broadly, Australia has recognised the need to support regions as it transitions away from a reliance on fossil-fuels, and its 2023 budget announced the establishment of a Powering the Regions Fund to support investment and innovation in regions in a decarbonising economy, including in bioenergy [161]. In Queensland, a much greater focus for regional development is embedded in policy, including the vision being that integrated biorefineries could create over 4000 jobs, predominantly in regional Queensland. The Queensland Biofutures Roadmap emphasises government-industry-research collaboration through Government supported hubs, precincts and incubators [112]. The planned Future Foods BioHub applies innovative biotechnology to create, manufacture and export new food products, proteins and extractives, with a view to building on Mackay's agricultural strengths, create job opportunities and complement existing industries [162]. While not specific to biorefining industries, the Resource Centre of Excellence in Mackay and Agribusiness Connect in Toowoomba are dedicated hubs for local businesses looking to integrate circular economy principles into their operations as is the proposed Smart Precinct NQ in Townsville [163]. Hubs are supported through the Manufacturing Hubs grant program, which assists regional manufacturers build capability [164].

Bioclusters are a feature of bioeconomy in the EU. The European Cluster Collaboration Platform lists 68 bioeconomy bioclusters and 90 biopharmaceuticals clusters across Europe [165]. Regional economic development is a clear objective of the Bioeconomy Strategy [121]. The European Bioeconomy Network lists 58 (current and concluded) projects targeting the potential of regional and local bio-based business, supporting the deployment of bio-based innovation at regional level, and promoting the active involvement of local actors to revive regional areas [166]. The Green ERA-Hub coordinates and reinforces cooperation and funding between agri-food and biotechnology sectors [167]. The Common Agriculture Policy allows Member States to set out interventions adapted to locality to promote the development of the bioeconomy in rural areas and support primary producers in their efforts to innovate and drive the bioeconomy [125]. The Bioeconomy Strategy Accelerator Toolkit guides decision-makers and stakeholders to develop their regional bioeconomy strategies, including through networking, capacity building and information dissemination for stakeholders and policy makers [168]. Although not specifically targeting biorefining, relevant agricultural incentives include the European Agricultural Fund for Rural Development [169], which finances agri-environment-climate measures, and the European Agricultural Guarantee Fund provides direct payments to farms based on sustainable farming methods [170].

### 4.5 Social change policies

Australia doesn't impose sustainability standards and certification for bioproducts, and the immaturity of the industry means there is no current value in developing alternative instruments to communicate the benefits of products to consumers. There is recognition within biorefining strategies more broadly (e.g., the Bioenergy Roadmap) that social license, education and a skilled workforce will be key components of industry expansion. The recently proposed Powering the Regions Fund aims to assist build a new energy workforce, committing $100 million to address growing skills demand in the sector. Queensland's Waste Management and Resource Recovery Strategy highlights that education for communities and industry will drive changes in consumer expectations, knowledge and behaviour [109]. The strategy recognises workforce training and upskilling as key to Queensland's ‘biofuture’.

Social change and education are key components of EU strategic frameworks and action plans for the bioeconomy, in particular workforce education and training and this is reflected in 34 (current and concluded) programs dedicated to this task (per The European Bioeconomy Network) [166]. There is, however, very limited market pull developed in specific policy targeting bioproducts, with no certification and labelling for bioproducts, limited public awareness initiatives or favourable purchase policies. The EC has proposed legislation on the Sustainable Product Policy Initiative [171], setting technical standards for sustainability, which would extend the Eco-design Directive [171] (setting requirements for product groups to improve circularity, energy performance and other environmental sustainability aspects) beyond energy to bioproducts. For businesses, the Corporate Sustainability Due Diligence and Amending Directive (2022) proposes robust legislative measures mandating large EU and non-EU companies to set obligations for actual and potential adverse impacts on the environment [172]. This legislation is expected to drive systematic change across value chains, moving resources away from activities that negatively impact climate change and thereby offering broad support for bioeconomy.

## 5 Conclusion

This paper's analysis demonstrates several key insights. Firstly, Australia's biorefining industries are considerably less advanced than the EU's and associated policy frameworks less developed. As a key component of bioeconomy, policies targeting the development of biorefining and bioproduct industries have been embedded in EU policy instruments, at least indirectly, since 2012. A bioproduct industry, including from a FLW feedstock, is expressly referenced within strategic instruments across multiple policy domains at EU level, some of which incorporate legally binding provisions. By comparison, although Section 4 highlights a relatively broad range of recent state policies directly targeting a bioproduct industry across multiple policy domains in Queensland, nation-wide strategic policy instruments are limited in Australia. In developing its own bioproduct industry, Australia should consider bolstering strategic policy leadership at national level, including through the implementation of binding instruments. This would serve to 1) create clear national objectives for a bioproduct industry (including meeting ‘nutrient extraction’ objectives in line with SDG Target 12.3); 2) mitigate the risk of inter-state policy tensions where different state policies are unaligned, at odds or ambiguous; and 3) lay the groundwork for necessary policy stability and coherence for a bioproduct industry.

A critical starting point for Australia will be reframing FLW strategic instruments so that bioproducts are included within the scope of interventions for meeting Target 12.3. Further, and in the absence of overarching bioeconomic objectives, consideration should be given to a specific strategy for bioproducts - akin to those dedicated to bioenergy and biofuels - embedding clear vision and intent for the industry. Additionally, there is scope for Australia to strengthen policy settings by including express reference to a bioproduct industry within its waste, circular economic, technology and agriculture strategies and action plans. There is similarly potential for Australia to integrate its FLW nutrient extraction objectives into strategic instruments across relevant policy domains beyond food waste (including emissions reduction, agtech and waste management), as Queensland has done at state-level. Strengthening strategic policy was an issue considered in the Inquiry into Food Security (2023)[173], the central recommendation of which was the formulation of a National Food Plan engaging the whole food system to achieve food security and related outcomes. Exploring this proposition through a FLW bioproduct lens, along with other pertinent recommendations from the inquiry (appointing a Minister for Food and a National Food Council, establishing regional food and jobs hubs, developing mechanisms to promote innovation in food production and creating circular economy), would hold significant value.

Secondly, policy support for bioproducts in the EU remains largely limited to R&D grants, standardisation and education, and outreach tools, compared with the bioenergy, biofuels and petrochemical sectors, which also benefit from binding economic instruments (e.g., quotas and tax incentives). This accords with the theory [96] that economic policy instruments aren't effective during the early stages of development until stability and confidence in the market has been established and commercial viability proven. As a critical policy lever for a bioproduct industry at an stage of technology readiness, the level of R&D funding and how it is targeted will significantly impact the pace at which an Australian FLW bioproduct industry can advance. Australia would best direct R&D to the optimisation of FLW bioproduct processes, with specific consideration given to the technical, economic, environmental and social implications of desired feedstocks. Feedstock quantification and location are important datasets for optimisation assessments but are lacking in Australia and need to be prioritised to support a FLW bioproduct industry. R&D also needs to focus on robust and flexible logistics systems (collection, transport, storage) for a FLW feedstock. While monitoring frameworks are a relatively new addition to the bioeconomy in the EU, Australia should heed insights from research literature indicating that a concerted effort to advance an industry would best benefit from early and coordinated monitoring frameworks, as key instruments for assessing the need for policy intervention.

Thirdly, current policy settings in the EU and Australia favour bioenergy and biofuels industries – where their more advanced industry status allows for economic policy instruments, clearer policy intent, coherence and stability. The failure to expressly prioritise bioproducts over bioenergy and biofuels in policy risks diverting FLW suitable for product-driven biorefining to the lower value destinations of energy and fuel. This is at odds with overarching circular economic principles and the food waste hierarchy prioritising high value bioproducts over bioenergy and biofuels in a cascaded framework. Establishing a more level playing field for bioproducts will be critical, but until technology readiness levels increase, effective and viable cascading policies will be difficult to implement. Levelling the playing field will in the first instance, require Australia to implement policy supporting an accelerated R&D agenda for bioproducts. In the medium-long term, the critical juncture for policymakers as the bioproduct industry advances, will be recognising when conditions are appropriate for economic policy instruments (e.g., quotas and targets for the production of bioproducts and taxes or bans on fossil-based products) to take effect, in order that the prioritisation of high-value products in a circular economy is mirrored by strong policy incentives to support those ambitions. Subsidies and incentives will be critical to supporting industry advance – to build market demand, enhance competition, stimulate bioeconomic growth and meet environmental targets. Removing fossil fuel subsidies and pricing the environmental damage of those industries must be a consideration for Australia in that process. Ensuring regulatory standards incentivise the use of waste over alternative biomass feedstocks will not only be important to meeting Target 12.3 but will also be significant to ensuring sustainable processes and mitigating the risk of challenges arising around competition for food and land use.

Finally, the EU has ensured the integration of regional economic development across its biorefining and associated policy frameworks, from which Australia can draw valuable lessons. That said, Australia's distinctive regional landscape will pose unique challenges for bioproduct industry development, viability and optimisation. Establishing or bolstering biorefining operations will likely present highly localised social, economic and environmental issues. These will require careful consideration, including context-specific analysis and targeted policy support on issues such as workforce education and training, tax incentivisation to attract investment, and community engagement to promote social license.

There are several noteworthy externalities impacting the development of a FLW bioproduct industry that are beyond the scope of this review. Covid-19, supply chain challenges and Russia's invasion of Ukraine have significantly impacted energy security and affordability in recent years, which has heavily influenced policy in energy sectors globally and which has flow-on effects for an emerging bioproduct industry. The major and complex challenges of climate change, emissions reduction targets, and creating a jobs market in renewable industry all have important and potentially competing political and policy impacts on advancing an industry. Assessing the impacts of these factors in the Australian industry and policy context will be important in determining which policy settings would best suit the Australian landscape. This taxonomy and comparative review identify the breadth of policy pathways available to Australia to advance a bioproduct industry from a FLW feedstock. The impact of specific policy levers will only become clear through additional investigation and modelling. We recommend further research on the features of the Australian industry context, including further interrogation of current state policies directly and indirectly impacting product-driven biorefining using a FLW, to identify optimal policy settings.

## CRediT authorship contribution statement

**Hannah Churton:** Writing – original draft, Methodology, Investigation, Conceptualization. **Bernadette K. McCabe:** Writing – original draft, Methodology, Investigation, Conceptualization.

## Declaration of competing interest

The authors declare that they have no known competing financial interests or personal relationships that could have appeared to influence the work reported in this paper.

## Footnotes

1 Biorefining processes are distinct from other recycling, upcycling and recovery processes that do not include refining processes. For example, biorefining does not include the manufacture of food products (e.g., soups, nut bars, juice and dried fruit) but can include the refining of pure components (e.g. vitamins, fibre, proteins) that are subsequently included in human or animal food products. Composting does not incorporate biorefining processes. Digestate and other nutrient by-products of anaerobic digestion processes are not considered high-value for the purposes of meeting SDG Target 12.3.

2 There is no universally accepted definition of FLW, with varying definitions adopted by different countries and jurisdictions (FAO, 2019). Across the spectrum of definitions adopted globally, distinctions between avoidable/unavoidable FLW are often made. [19] defines unavoidable wastes as inedible parts of post-harvest food like fruit cores, peelings, and bones while possibly avoidable can include peels from potato or carrot as it can be eaten or used in processing, depending on the process specifics, consumer preference and regional regulations. This becomes significant when assessing the availability of feedstock - unavoidable and inedible FLW will persevere despite broader efforts to minimize FLW.
