# Supplementary material for: Advancing a food loss and waste bioproduct industry: A critical review of policy approaches for application in an Australian context
Source: Heliyon. 2024 Jun 9;10(12):e32735. doi: 10.1016/j.heliyon.2024.e32735 (PMC11225737; doi:10.1016/j.heliyon.2024.e32735)
Supplement: Multimedia component 1 [file mmc1.rtf]

From: "JCLP" <em@editorialmanager.com>Subject: Decision on submission to Journal of Cleaner ProductionDate: 22 November 2023 at 5:28:25 m AESTTo: "Hannah Churton" <hannah.churton@hdr.qut.edu.au>Reply-To: "JCLP" <support@elsevier.com>Manuscript Number: JCLEPRO-D-23-27668   dvancing a food loss and waste bioproduct industry: A critical review of policy approaches for application in an Australian context     ear Ms hurton,      hank you for considering Journal of Cleaner Production.  e have examined your manuscript carefully; however, we have decided it is not the right fit for our journal.  n support of your publication journey, please see the below message from our Scientific Manging Editor, who has identified potential journals for your consideration as part of our Article Transfer Service.   hank you, again, for giving us the privilege of considering your article. We appreciate your support of Journal of Cleaner Production and warmly welcome your future breakthrough submissions.   incerely,   r. Cecilia Maria Villas Bôas de almeida  *********  s Dr. almeida has mentioned, in my role as Scientific Managing Editor and transfer liaison with our partner journals, I have further reviewed your submission.   am pleased to offer a conditional acceptance of your manuscript in the relevant section of Heliyon, an all-science, open access journal publishing scientifically valuable research. Heliyon is part of Elsevier’s prestigious Cell Press and has an Impact Factor of 4.0. www.cell.com/heliyon  f you take up this offer and transfer your submission to Heliyon, your paper will be quickly accepted, without further peer review. Please note that acceptance is contingent upon your revising your manuscript to address the comments of the reviewers and editor, which follow below.  o take advantage of this conditional acceptance offer, simply click the link below by Mar 31, 2024. You will then have 90 days to finalise your submission to Heliyon. During this stage, please upload a tracked-change revision of your manuscript as well as a comprehensive "Response to Reviewers” confirming that the required revisions have been implemented.  gree to Transfer  lternatively, given the merits of your research and the topics covered by your paper, while I cannot offer conditional acceptance in the following journal(s), you may wish to submit your manuscript there for consideration. Please note that your paper will proceed through the standard editorial process. eliyon	https://www.editorialmanager.com/HELIYON/	
Sustainable Futures	https://www.editorialmanager.com/SFTR/	
Sustainable Futures (Impact Factor 5.5, CiteScore 6.5) is an open access peer-reviewed journal focused on sustainability research in all areas of the social sciences. It provides an advanced platform for studies related to sustainability and sustainable development in society, economics, environment, and culture. The scope of the journal is broad and encourages interdisciplinary research, as well as welcoming theoretical and practical research from all methodological approaches. If you choose to transfer your manuscript to this journal before December 2024 and if your article is accepted in that journal for publication, then you will be eligible to receive an APC reduction of 100%. For more information, please visit: https://www.journals.elsevier.com/sustainable-futures  o transfer your paper, please click the link below by Mar 31, 2024. You will also have 90 days to finalise your submission at your preferred journal.  gree to Transfer  f you have any questions about the transfer of your paper, please do not hesitate to contact me directly.  ind regards,  wathi Desireddy, Ph.D. cientific Managing Editor .desireddy@elsevier.com  ournal of Cleaner Production    ditor and Reviewer Comments (if applicable):     E Comments:  hank you for considering Journal of Cleaner Production.  fter careful review, I regret to inform you that reviewers don't support to publish your above noted manuscript for publication in "Journal of Cleaner Production". I have appended the comments of the reviewers in order for you to understand the basis for the final decision.   eviewer #1: The study attempts to conduct a comprehensive systematic review on policy approaches for application of advancing FLW bioproduct industry, which is an interesting topic and worthy of investigation. Anyway, I do not think the manuscript is not good enough to publish in JCLEP. irst, some errors need to be corrected, such as the Key Concepts section, which should be section 2 but not 1. Moreover, this section is a little weird and not necessary to be placed here as a section. I'd suggest the authors to combine it into other section. econd, I am a little confused about the definition of "policy". What policy should include? In the manuscript, research and development grants is also considered as policy. In my opinion, the research should narrow the meanings of policy and focuses on certain policies especially incentives, subsidies and taxes. hird, I am still not clear on the policy framework of Australia on food loss and waste bioproduct industry after reading the manuscript. The study talked a lot on the comparation between Australia and EU. The reason of choosing EU as benchmark is not provided. ourth, the logic integrity of the manuscript is not so sound.   eviewer #2: The paper deliberates on the food loss and waste industry in the Australian context. The paper reads generally well. However, I have a few observations to improve the quality of the script.  ajor observations:  . In the introduction, it would be good to have some examples of bioproducts of food loss and waste (in general and Australian context). . Figure 1 can be shifted from the introduction to subsequent sections. . Towards the end of the introduction section, I expect the authors to discuss the key contributions of your research. . The entire paper stands on bioproducts, I would prefer to read a complete discussion on that in Section 1. t takes only 6 lines in Section 1, Key Concepts. This section has many subsections - no connections/transitions are also not smooth. How about combining everything into one paragraph and starting straight into bioproducts in the next subsection . Section 3- How about structuring with the importance of bioproducts and how this industry is evolving - this section has to be strengthened. . In 3.2.8 - Policy suability and coherence - it is necessary to bring the cases on how governance plays a key role and link it with the UN-SDG pointers. . Figure 2 needs a complete revision- as I see the overlap between institutional policy and social policy framework - It needs a better rationalization on those proposed five pillars. I see, they need to start with global then integrate the Australian context here, I understand that they were trying to do that 5.2 & 5.3 - but adding these (Australian) perspectives helps readers to relate it better. lso, I invite authors to propose some propositions or strategic frameworks apart from taxonomy. . I expect the authors to propose the theoretical contributions of the study. The draft needs one.  inor  Check the section numbering.  Reading flow is missing in Sections 2 & 3.   eviewer #3: This is a well-written paper that identifies some notable differences between the Australian and EU approach and explains the implications of these differences. While I think the paper is broadly very good, I do not think it quite suits the Journal of Cleaner Production. I think it would be better suited in another journal. While the paper makes interesting and useful points, it is not quite significant enough, in my opinion, to be in this particular journal, combined with the lack of clear methods. This is a great literature review, which is very hard to achieve, but it is not an empirical analysis nor does it clearly explain the methods used other than literature review by seemingly one author (e.g.. in a systematic review, it would involve particular steps and checks along the way/qualitative documentary analysis also has particular requirements). It also is not making highly novel points, which is fine not all papers need to do that, but I think for this journal they should be in that ballpark. To be published just as a literature review in this journal, I would suggest, the findings would have to be persuasively presented as highly important. In this case, I do not think the findings are original or wide-reaching enough - that's not to say that they're not important and shouldn't be published elsewhere. As an aside, the key concepts section at the start is not needed given the audience/context for this journal and simply some concepts can be said without explanation or said with a brief explanation as they arise. Also, the explanation of how the taxonomy was developed was very brief. I really think this piece would be great in another journal that was perhaps a bit more accessible. I can see how this piece would be highly interesting to respective government bodies, so I hope when the author does publish it that they also share it with relevant government actors. Clearly, there's a lot of potential for future work in this area to go into this journal.          ore information and support     When the transfer of your manuscript is complete (usually within 48 hours) you will receive an email containing further instructions on how to complete your submission.    You will be able to revise your manuscript prior to resubmission. When you submit your revised manuscript, please ensure you include a cover letter detailing all changes to the manuscript, as well as a "Response to Reviewers" containing all reviewer comments and a response to each comment.    You will find more information about Elsevier's Article Transfer Service here:  ww.elsevier.com/authors/article-transfer-service.  ou will find information relevant for you as an author on Elsevier’s Author Hub: https://www.elsevier.com/authors   AQ: How can I reset a forgotten password?  ttps://service.elsevier.com/app/answers/detail/a_id/28452/supporthub/publishing/  or further assistance, please visit our customer service site: https://service.elsevier.com/app/home/supporthub/publishing/ ere you can search for solutions on a range of topics, find answers to frequently asked questions, and learn more about Editorial Manager via interactive tutorials. You can also talk 24/7 to our customer support team by phone and 24/7 by live chat and email   t Elsevier, we want to help all our authors to stay safe when publishing. Please be aware of fraudulent messages requesting money in return for the publication of your paper. If you are publishing open access with Elsevier, bear in mind that we will never request payment before the paper has been accepted. We have prepared some guidelines (https://www.elsevier.com/connect/authors-update/seven-top-tips-on-stopping-apc-scams ) that you may find helpful, including a short video on Identifying fake acceptance letters (https://www.youtube.com/watch?v=o5l8thD9XtE ). Please remember that you can contact Elsevier s Researcher Support team (https://service.elsevier.com/app/home/supporthub/publishing/) at any time if you have questions about your manuscript, and you can log into Editorial Manager to check the status of your manuscript (https://service.elsevier.com/app/answers/detail/a_id/29155/c/10530/supporthub/publishing/kw/status/).In compliance with data protection regulations, you may request that we remove your personal registration details at any time. (Remove my information/details). Please contact the publication office if you have any questions.
